# A comparison of global mangrove maps: Assessing spatial and bioclimatic discrepancies at poleward range limits

**DOI:** 10.1016/j.scitotenv.2022.160380

**Published:** 2023-02-20

**Authors:** Arimatéa C. Ximenes, Kyle C. Cavanaugh, Damien Arvor, Daniel Murdiyarso, Nathan Thomas, Gustavo F.B. Arcoverde, Polyanna da Conceição Bispo, Tom Van der Stocken

**Affiliations:** a Center for International Forestry Research (CIFOR), Jl., Situgede, Bogor 16115, Indonesia; b Department of Geography, University of California Los Angeles, Los Angeles, USA; c CNRS, UMR 6554 LETG, Université Rennes 2, 35043, Rennes, France; d Earth System Science Interdisciplinary Center, University of Maryland, College Park, MD 20740, USA; e Biospheric Sciences Laboratory, NASA Goddard Space Flight Center, Greenbelt, MD 20771, USA; f National Institute for Space and Research - INPE,São José dos Campos, Brazil; g Department of Geography, School of Environment, Education and Development, University of Manchester, Oxford Rd, Manchester M13 9PL, UK; h Laboratory of Plant Biology and Nature Management, Ecology & Biodiversity, Vrije Universiteit Brussel, Brussels, Belgium; i Department of Geophysics and Meteorology, IPB University, Bogor 16680, Indonesia

**Keywords:** Species distribution, Range limits, Climate change, Coastal wetland, Mapping

## Abstract

Mangrove distribution maps are used for a variety of applications, ranging from estimates of mangrove extent, deforestation rates, quantify carbon stocks, to modelling response to climate change. There are multiple mangrove distribution datasets, which were derived from different remote sensing data and classification methods, and so there are some discrepancies among these datasets, especially with respect to the locations of their range limits. We investigate the latitudinal discrepancies in poleward mangrove range limits represented by these datasets and how these differences translate climatologically considering factors known to control mangrove distributions. We compare four widely used global mangrove distribution maps - the World Atlas of Mangroves, the World Atlas of Mangroves 2, the Global Distribution of Mangroves, the Global Mangrove Watch. We examine differences in climate among 21 range limit positions by analysing a set of bioclimatic variables that have been commonly related to the distribution of mangroves. Global mangrove maps show important discrepancies in the position of poleward range limits. Latitudinal differences between mangrove range limits in the datasets exceed 5°, 7° and 10° in western North America, western Australia and northern West Africa, respectively. In some range limit areas, such as Japan, discrepancies in the position of mangrove range limits in different datasets correspond to differences exceeding 600 mm in annual precipitation and > 10 °C in the minimum temperature of the coldest month. We conclude that dissimilarities in mapping mangrove range limits in different parts of the world can jeopardise inferences of climatic thresholds. We expect that global mapping efforts should prioritise the position of range limits with greater accuracy, ideally combining data from field-based surveys and very high-resolution remote sensing data. An accurate representation of range limits will contribute to better predicting mangrove range dynamics and shifts in response to climate change.

## Highlights

• Accurately measuring range limits is essential to monitoring mangrove expansion.
• Global maps differ in the locations of mangrove range limits.
• These differences will impact models of mangrove distributions and their response to climate change.

## Graphical abstract

**Figure:**
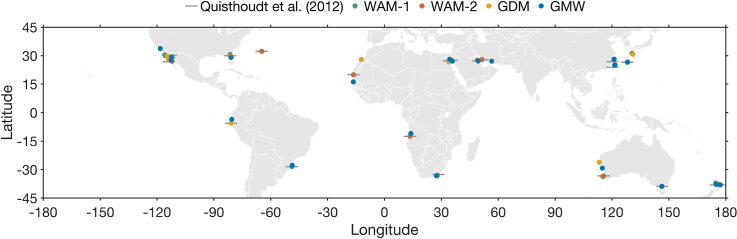
Global map showing the position of poleward mangrove range limits in 21 regions, as identified in four widely used global mangrove distribution maps and as identified by Quisthoudt et al. (2012), which is considered as a reference position of range limits in our study.

## 1 Introduction

The range limits of a species or ecosystem represent the ecomorphological edges and the environmental and climatic limits that constrain it (Thomas, 2010). These locations are often near the environmental tolerance threshold of the species or ecosystem and so they are critical to understanding responses to changes in environmental and climatic conditions (Cavanaugh et al., 2019; Ximenes et al., 2021). These regions are often defined as transition regions, where colonisation and primary growth are most apparent and where land cover is most sensitive to change. Global ecosystem extent maps are used and more–so often required for understanding changes induced by future climate predictions. While useful for this, they must be used with caution with an understanding of their caveats. As global extent maps are used to define the climatic variables that control ecosystem extent in climate–response models, results are heavily dependent upon the representation of range limits. Therefore, it is imperative that an ecosystem's range limits, while small in extent, are accurately and appropriately represented in global maps in order to derive a complete and accurate understanding of their response to global-scale processes of change.

Mangroves are one such ecosystem that have a broad distribution with climatically sensitive range limits. They are halophytic intertidal vegetation, most commonly represented by shrubs and trees, at the sea–land interface and distributed worldwide on tropical and subtropical shorelines (Tomlinson, 2016). They provide a broad range of valuable ecosystem services such as food provisioning, timber, fuel wood, coastal protection, erosion control, and habitat provision for fisheries (Barbier et al., 2011). In addition, they sequester disproportionate amounts of carbon for their area coverage and are considered important long-term carbon sinks (Donato et al., 2011; Alongi, 2014); a capacity and role that has drawn increasing attention in the context of climate–change mitigation (Murdiyarso et al., 2015; Taillardat et al., 2018). Yet, despite their ecological, societal, and economical importance, mangroves have been threatened by human activities, particularly land conversion for aquaculture, agriculture and urban development (Richards and Friess, 2016), as well as pollution (Duke, 2016).

Due to both natural processes and human activities, mangroves are very dynamic ecosystems, whose mapping and monitoring is challenging. Maps are designed to help visualise and comprehend landscapes where a systematic planning of natural resources and area estimates of certain habitats need to be carried out (Turner et al., 2003). For this reason, maps are essential to estimate deforested and degraded areas (FAO, 2003, FAO, 2007) and design protected areas and actions to ensure efficient conservation of mangroves. In this regard, accurate geographical distribution maps of mangroves are crucial to monitor the spatial and temporal variability in mangrove forest extent and better understand the environmental and human drivers of these changes (Cavanaugh et al., 2014; Thomas et al., 2017; Osland et al., 2017b; Cavanaugh et al., 2018; Goldberg et al., 2020; Worthington et al., 2020). In addition, accurate representations of global mangrove extent may reduce uncertainties in biomass and carbon stock assessments (Simard et al., 2019; Rovai et al., 2021) which is important to inform and support mitigation and adaptation policies. Over recent decades, the potential of remote sensing techniques to identify and map mangrove forests has been extensively researched (Satyanarayana et al., 2011; Diniz et al., 2019; Simard et al., 2019; Pham et al., 2019; Valderrama-Landeros et al., 2021). A number of studies have used the global mangrove maps to locate mangrove range limits and model how mangrove distributions may be impacted by climate change (Quisthoudt et al., 2012; Osland et al., 2017b). However, small differences in the location of mangrove range limits in the datasets could influence the climatic thresholds associated with mangrove presence and absence (Cavanaugh et al., 2015; Ximenes et al., 2021). Despite these issues, global maps are widely used by the scientific community, but the advantages and limitations of available global products have never been compared and discussed. A new initiative in this matter allows users to compare global maps of mangrove extent, biomass and carbon (see further information in Section 4.3).

To date, four global maps of mangroves have been produced and released publicly (Spalding et al., 1997, Spalding et al., 2010; Giri et al., 2011; Bunting et al., 2018). These global maps cover different time periods and were derived using different datasets and methods. As a result, there are differences that may be due to changes in actual mangrove extent, mapping error and differences due to methods and datasets used (Bunting et al., 2018). While these maps have been validated using published records of mangrove presence, challenges associated with conducting comprehensive global accuracy assessments make it difficult to quantitatively compare the performance across maps. Also, the accuracy of each map suffers from spatial heterogeneity where regional mapping quality varies and is represented by a global statistic of accuracy alone. Hence, we need a better understanding of the differences in these datasets in order to understand mangrove response to future climatic perturbations and modelling.

In particular, the correct location of each poleward mangrove range limit is crucial to understand the climatic drivers or range limitation and project the impacts of climate change. There are at least 21 poleward mangrove range limits Quisthoudt et al. (2012) and the correct mapping of these mangroves at their range limits is challenging. Due to extreme climate conditions at these locations, the mangrove trees are usually smaller in structure, lower density, and smaller in extent as compared with their counterparts closer to the equator. For this reason, identifying errors in the geographical location of the mangrove range limits is fundamental for future mapping efforts.

Here, we present the first comparative study for global mangrove datasets with a clear focus on range limits worldwide and identify potential discrepancies between these products. We investigate differences in latitudinal range limits between the four global datasets and how these discrepancies translate climatologically considering factors known to control mangrove distributions. Based on our observations and climate data analyses, we formulate recommendations to inform the future production of global mangrove maps.

## 2 Material and methods

The global mangrove maps considered in this study: (1) the World Atlas of Mangroves (WAM-1) Spalding et al. (1997), (2) the World Atlas of Mangroves (WAM-2) Spalding et al. (2010), (3) the Global Distribution of Mangroves (GDM) Giri et al. (2011) and (4) the Global Mangrove Watch (GMW) Bunting et al. (2018). These maps are made available at: https://data.unep-wcmc.org/. The main characteristics of these different products are summarised in Table 1 and detailed in the following subsections.

**Table 1:** General information of the four global mangrove maps. The metadata of the four global mangrove maps were based on: spatial resolution, period (time), Sensors, Methods, reference and data access.

Product	WAM-1	WAM-2	GDM	GMW
Resolution	Various	30 m and higher resolutions from 1999 to 2003	30 m from 1997 to 2000	30 m 2010
Period	Various			
Sensors	NOAA-AVHRR SPOT HRV LANDSAT 4 MSS LANDSAT 5 TM ERS-1	Landsat5-TM Landsat7 ETM+ ETOPO1-NOAA SRTM	LANDSAT 5 TM LANDSAT 7 ETM+	LANDSAT 5 TM LANDSAT 7 ETM+ ALOS PALSAR
Method	Manual delineation	Unsupervised classification with edition of results	Hybrid supervised and unsupervised classifications	Extremely randomized trees classification
Reference	Spalding et al. (1997)	Spalding et al. (2010)	Giri et al. (2011)	Bunting et al. (2018)

It is worth noting that the Global Database of Continuous Mangrove Forest Cover for the 21st Century (CGMFC-21) (Hamilton and Casey, 2016) was not included in this paper because it used the GDM map as a reference of mangrove mapping. Therefore, although the CGMFC-21 map is more restrictive in its definition of mangroves (the total mangrove area in CGMC-21 is 39 % smaller than in the GDM map), the CGMFC-21 map and the GDM map are spatially correlated.

### 2.1 Global mangrove maps

#### 2.1.1 World Atlas of Mangroves-1 (WAM-1)

The first World Atlas of Mangroves (WAM-1) was launched by the World Conservation Monitoring Centre (WCMC) and released in 1997 (Table 1). The starting point to map the mangrove coverage for the WAM-1 was originally taken from *The Conservation Atlas of Tropical Forests* which involved several organisations, governments, agencies, and scientists (Spalding et al., 1997). Identifying gaps, updating obsolete data, improving low resolution data, and adding new datasets were done through correspondence and discussion with many authorities on this topic. In addition, the WAM-1 used satellite images acquired at different dates and spatial resolutions such as: the National Oceanic and Atmospheric Administration/Advanced Very High Resolution Radiometer (NOAA-AVHRR), the Land Remote Sensing Satellite Program (LANDSAT) - Mutispectral Scanner (MSS) and Thematic Mapper (TM) sensors, the Satellite Pour l'Observation de la Terre - High Resolution Visible sensor (SPOT-HRV), and the European Remote Sensing Satellite (ERS-1 and 2) (Spalding et al., 1997). Aerial photographs were also used at some specific locations (Spalding et al., 1997). The WAM-1 map was hand-drawn by experts visually delineating mangrove areas in remote sensing images (Spalding et al., 1997).

The authors of WAM-1 claimed that the different spatial resolutions of satellite images used in this mapping may determine differences in spatial accuracy. For instance, even large areas of mangrove patches can be omitted if they are narrow and therefore difficult to recognise in low-resolution images.

#### 2.1.2 World Atlas of Mangroves-2 (WAM-2)

The second World Atlas of Mangroves (WAM-2) was reformulated from the WAM-1 and was published in 2009, twelve years later (Spalding et al., 2010). The WAM-2 was led by the Food and Agriculture Organization of the United Nations (FAO) and the United Nations Environment Programme – World Conservation Monitoring Centre (UNEP- WCMC). The WAM-2 map improvements over the WAM-1 were mainly: (i) gather higher spatial resolution images for nearly all mangrove areas globally; (ii) mapping improvements with 98.6 % of the total global mangrove area coverage mapped by the WAM-1. To assist the production of the WAM-2 map, four datasets in particular were considered: (i) Topography and Bathymetry – extracted from SRTM and ETOPO1 global relief model; (ii) Populated places, rivers and lakes – derived from Global Rivers database; (iii) Coastal geographical features – extract from World Vector Shoreline; (iv) Protected areas – provided by UNEP-WCMC and produced by World Bank Database on Protected Areas.

The WAM-2 was built using various techniques, including the selection of classes from unsupervised classifications of remote sensing images, the use of a digital elevation model to exclude unsuitable sites for mangroves, secondary sources of mappings, visual interpretation by local field experts and geographic context data layers to assist producing the final map – i.e., populated places, rivers and lakes, coastal geographical features, and protected areas (Spalding et al., 2010).

UNEP-WCM began to map mangroves in several countries for which data were available. FAO prioritised countries where the 1997 World Mangrove Atlas data was outdated. UNEP-WCMC built a geodatabase mainly using the Landsat 5 TM and Landsat 7 ETM+ images, dating predominantly from 1999 to 2001. The satellite images used in the mapping were composed by band 5 (Short-Wave Infrared - SWIR), band 4 (Near Infrared - NIR), and band 3 (Red) to supply semi-automatic classifications. The image classifications were carried out according to: (i) image geometry and radiometric corrections, (ii) visual interpretation, (iii) unsupervised classification, (iv) review of results, (v) editing and (vi) external review (Spalding et al., 2010).

The pre-classification was performed after spatial, spectral and radiometric image correction. The visual interpretations were used to select potential mangrove areas as regions of interest (ROI) for semi-automatic classifications. The unsupervised classification found 20 clusters of which four were selected as the best mangrove spectral pattern. The selected four classes were edited using Shuttle Radar Topography Mission (SRTM) images to assist the visual interpretations. Several experts from different regions globally (mainly from African countries) provided visual interpretations to increase the level of confidence of the mapping (Spalding et al., 2010).

Between the years 1999 and 2003, FAO worked with Landsat ETM+ images and secondary mapping sources. Landsat images were used for visual interpretation and compositions of spectral image bands were used to enhance mangrove stands at 1:250.000 scale. To review the visual interpretation from WAM-1, other partners – the International Society for Mangrove Ecosystems (ISME) and the International Tropical Timber Organization (ITTO) and UNEP-WCMC – assisted by local experts to improve digitisation of particular sites. This methodology covered 57 % of the global mangrove area (86,000 km^2^). The second methodology was mapped at 1:250.000 scale, which came from several institutions: FAO for African and Red Sea coastline, The Nature Conservancy (TNC) mainly responsible for the Caribbean, the Central America and the Pacific region, and National data from approx. 20 countries and territories. This methodological consortium covered about 41,7 % of the global mangrove area (63,000 km^2^). Both mapping methodologies led to several overlaid maps. For this reason, the resulting maps were reviewed by specialists and by technical staff at FAO to produce reliable map layers by country.

#### 2.1.3 Global Distribution of Mangroves (GDM)

The Global Distribution of Mangroves (GDM) map was produced by the United States Geological Survey (USGS) team (Giri et al., 2011). The GDM map is based on the Global Land Survey (GLS) data, i.e., Landsat images prepared in partnership between the USGS and the National Aeronautics and Space Administration (NASA). For this mapping, a global dataset of Landsat 5 Thematic Mapper (TM) with 30 m of spatial resolution acquired from 1997 to 2000 (Giri et al., 2011) was used. About a thousand Landsat images were interpreted using hybrid supervised and unsupervised digital classification techniques to estimate and map the total area of global mangrove forests (Giri et al., 2011). Moreover, the global mangrove database from FAO (2007) and national and local mangrove database were used as secondary data (Giri et al., 2011).

Pre-processing of images consisted of a geometric correction to improve the geolocation to a root mean square error of half a pixel, a normalisation of the images for variation in solar angle and earth-sun distance, and excluding the thermal band (band 6) (Giri et al., 2011). The authors reported that a robust global validation was not available, so they relied on the help of local experts and high-resolution satellite images available in Google Earth to perform qualitative validation. A supervised classification was done to map water bodies, and subsequently, an ISODATA clustering algorithm was applied (Giri et al., 2011). From the clustering, four classes were generated: mangrove, non-mangrove, barren lands and water bodies. The definition considered for ‘true mangrove’ was from Tomlinson (2016) and encompasses trees, shrubs and palms that grow exclusively in the tidal and inter-tidal zones of the tropical and subtropical regions.

#### 2.1.4 Global Mangrove Watch (GMW)

The Global Mangrove Watch (GMW) map is the most recent mapping initiative of mangrove ecosystems and is part of the Japan Aerospace Exploration Agency (JAXA) Kyoto & Carbon Initiative with the objective to generate a global map of mangroves for the year 2010. GMW takes full advantage of combining optical and SAR (Synthetic Aperture RADAR) images (Bunting et al., 2018) and relies on ALOS PALSAR L-band SAR dual polarisation (HH + HV) backscatter data released in 1° × 1° mosaic tiles (Shimada et al., 2014) to discriminate mangroves. Since some confusions with other wetland or forest types remained, the near infrared and shortwave infrared band of Landsat data (optical) were used to reduce the confusion between these land cover classes (Bunting et al., 2018).

Currently, the GMW map is led by Aberystwyth University (U.K.) and Solo Earth Observation (Sweden). The GMW map was developed in collaboration with Wetlands International, the International Water Management Institute (Laos) and the United Nations Environment Programme (UNEP) World Conservation Monitoring Centre (U.K.).

The methodology used to produce the GMW mangrove for 2010 involved a combination of ALOS PALSAR and optical satellite data from Landsat 5 Thematic Mapper (TM) and Landsat-7 Enhanced TM (ETM+). The authors used the composite images from ALOS PALSAR of the 2010 mosaic as a reference mainly because it was the most complete in terms of temporal consistency and spatial coverage. A composite was also generated using Landsat sensor data acquired for 2010 mainly. Bunting et al. (2018) used four main methodological steps to produce the GMW mangrove extent which included (i) the extraction of a coastal water mask from the PALSAR data; (ii) generating a mangrove “habitat” layer that identified areas potentially able to support mangroves; (iii) generating an initial baseline classification using the PALSAR data only; and (iv) a refinement of the classification using Landsat sensor composites. A final quality assessment was undertaken to identify and correct any potential errors and inaccuracies. More details about the methodology can be found in Bunting et al. (2018).

### 2.2 Mangrove range limits: latitudinal position and reference

We identified the latitude for twenty-one mangrove range limits in the four global datasets (see Supplementary material). As a reference, we used the twenty-one mangrove range limit positions considered by Quisthoudt et al. (2012) because these latitudinal limits were gathered from the literature and verified through communication with local mangrove specialists. Also, their study focused on mangrove latitudinal limits of *Rhizophora* and *Avicennia*, the only two pantropical mangrove genera of which species are generally found at all mangrove range limits around the world. Hence, this approach allows comparing the latitudinal position of mangrove range limit sites globally. For each of the twenty-one mangrove range limit sites, we computed the latitudinal difference between the reference dataset Quisthoudt et al. (2012) and the four mangrove maps. Since the global mangrove maps do not differentiate species, we used the most poleward location for each range limit in the Quisthoudt et al. (2012) data, regardless of whether it was *Rhizophora* or *Avicennia*.

#### 2.2.1 Bioclimatic variables at mangrove range limits

For each of the range limits presented in the different mangrove maps, we examined differences in the values of bioclimatic variables related to the distribution of mangroves. Environmental data were obtained from the WorldClim Version 2.1 database (Fick and Hijmans, 2017) freely available at: www.worldclim.org, which consists of spatially high-resolution (approximately 1 km^2^ at equator) raster layers of climate and are the average for the years 1970–2000. This historical climate data can vary regarding the availability of each local meteorological station (Fick and Hijmans, 2017). We focused on minimum air temperature of the coldest month (BIO6), annual precipitation (BIO12), and precipitation of the driest month (BIO14), since these three variables have been put forward as playing an important role in determining mangrove latitudinal limits (Quisthoudt et al., 2012; Cavanaugh et al., 2014; Ximenes et al., 2016; Osland et al., 2017a). The “Min temperature of coldest month” or BIO6 is a multi-decade average of the minima of the coldest months, which is therefore comparable to the “mean temperature of the coldest month” (Hijmans et al., 2005). In this study, we use the minimum temperature of the coldest month as a proxy of extreme cold events. However, the intensity, frequency, and duration of extreme events from hourly and/or daily climate measurements (i.e., the intensity, duration, and frequency of the absolute coldest temperatures of the year) is not available or hardly accessible statistic in a global database. However, it is the extreme cold events, rather than the mean, which periodically halt the poleward expansion of mangroves (Osland et al., 2017a).

Bioclimatic variables from the WorldClim database were generated for the terrestrial realm, so that the variable files contain ‘no data’ in the marine realm. However, mangroves thrive at the ocean-land interface, and some mangrove patches are positioned in marine ‘no data’ locations. For this reason, a land-ocean mask was generated with the same size and resolution as the bioclimatic data, and range limit longitude-latitude information from all mangrove maps were updated to the center of the nearest land grid cell. The processing of the land-ocean mask and figures related to this part of the study were generated using MATLAB version R2020b (MathWorks, 2020). Subsequently, using the updated longitude-latitude information, corresponding bioclimatic data were extracted at the above mentioned twenty-one mangrove range limits for all mangrove datasets. Data extraction was performed using the QGIS 3.14.0 software (QGIS Development Team, 2020).

## 3 Results

### 3.1 Global mangrove mapping characteristics

The four global mangrove maps vary in important aspects, such as the number of countries and territories where mangroves are observed, total mangrove area, number of mangrove polygons, as well as digital storage and global polygon mean area (Table 2). The file size and number of polygons vary greatly between all maps. The WAM-1 vector file has the smallest size, however with less details compared to more recent maps and less mapped countries and territories. The WAM-1 map contains much fewer polygons than more recent maps, e.g., at least 40 times fewer polygons than the GDM map. The main reason for this increase in disk storage is due to the use of higher spatial resolution satellite images and a higher accuracy in the latest mangrove maps (Table 2).

**Table 2:** Comparison among the four global maps. The “Global polygons mean area” is the total mangrove area divided by the number of polygons in the dataset, with smaller mean values representing more fragmented mangrove patches in the map.

Mangrove maps	WAM-1	WAM-2	GDM	GMW
Total mangrove area (km^2^)	181,077	152,000	137,760	137,600
No of countries and territories	112	123	118	108
No of polygons	34,315	1,115,610	1,397,008	496,555
Digital Storage (GB)	0.0407	0.854	1.18	0.839
Global polygons mean area (km^2^)	5.28	0.14	0.10	0.28

The number of polygons strongly increased for the two most recent mangrove extent maps compared to WAM-1. The GDM map estimates the total area of mangrove forests to be approximately 10 % smaller than reported in WAM-2 (Table 2). All global extent maps show mangroves in more than one hundred countries and territories worldwide (Spalding et al., 1997, Spalding et al., 2010; Giri et al., 2011; Bunting et al., 2018).

**Fig. 1:**
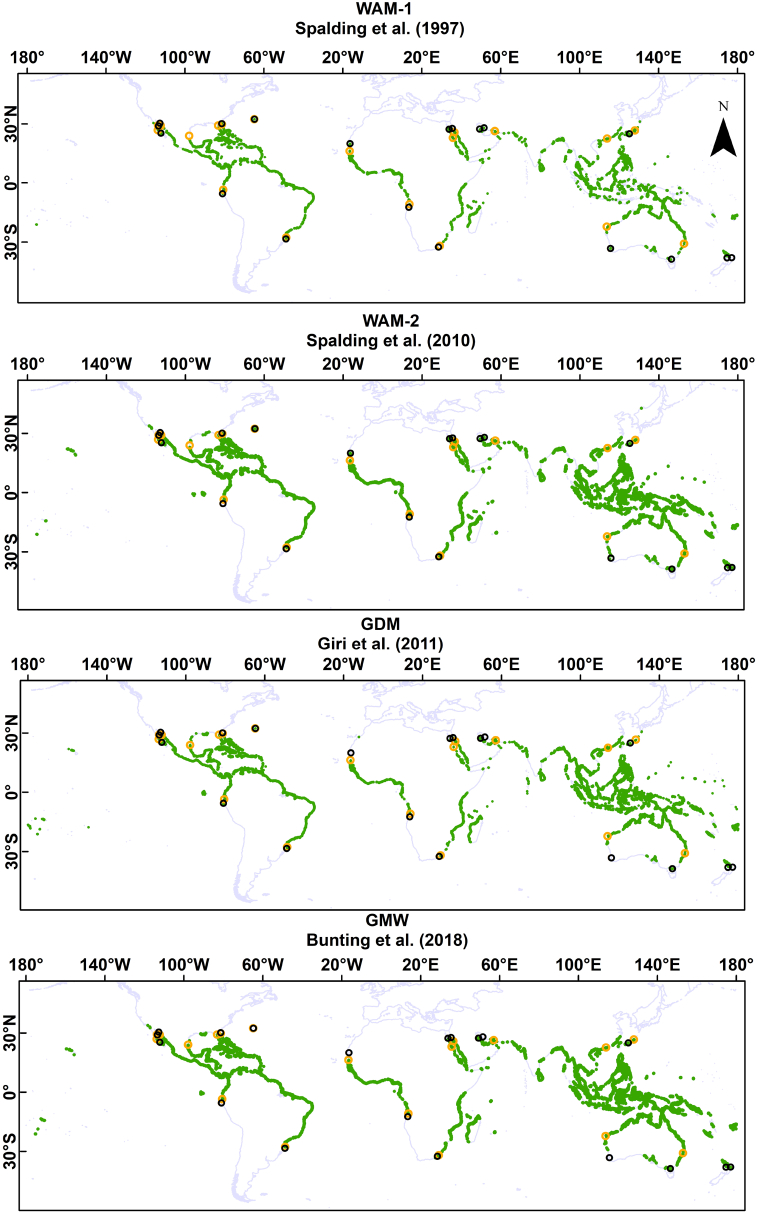
The four global mangrove extent maps considered in this study with the *Avicennia* sp. (black circles) and *Rhizophora* sp. (orange circles) range limits from (Quisthoudt et al., 2012). In total, twenty-one worldwide mangrove range limits were considered: (i) Western Baja California, (ii) Eastern Baja California, (iii) Sonora, (iv) Western South America, (v) Eastern North America, (vi) Eastern South America, (vii) Bermuda, (viii) Northern West Africa, (iv) Southern West Africa, (x) Northern East Africa, (xi) Southern East Africa, (xii) Western Saudi-Arabia (Red Sea), (xiii) Eastern Saudi-Arabia (Persian Gulf), (xiv) Iran, (xv) China, (xvi) Taiwan, (xvii) Japan, (xviii) West-Australia, (xix) East-Australia, (xx) Western New Zealand, (xxi) Eastern New Zealand. These maps were generated using the ArcGIS Desktop version 10.5 (ESRI, 2011).

For oceanic islands (mainly in the Pacific Ocean), the GDM map is much more spatially extensive compared to the other mangrove maps, despite that it is lacking mangroves along the coast of La Réunion (see Fig. 1). The oceanic islands are generally well mapped in the GMW map and WAM-2; however, some Pacific Island mangroves are missing. Regarding the ocean islands, the WAM-1 was found to be less detailed than other maps. Important improvements can be observed for the WAM-2 map compared to the older WAM-1 version, particularly in the Pacific Island Countries and territories of the Western and Central Pacific and Papua New Guinea.

The global polygons mean area, calculated as the total mangrove area divided by the total number of polygons, is a measure of the fragmentation of mangrove patches. The GDM map showed with smaller polygons mean area, thus being the most fragmented map followed by WAM-2, GMW and WAM-1, respectively (Table 2).

### 3.2 Latitudinal comparison between mangrove range limits

**Fig. 2:**
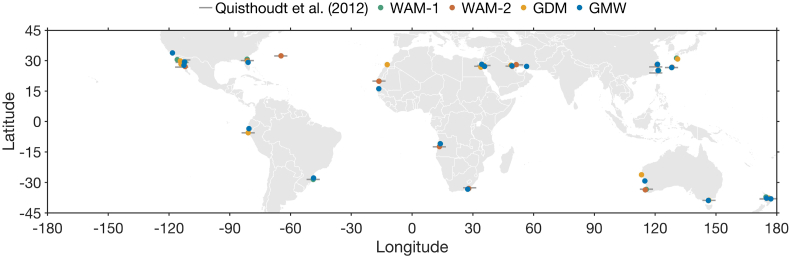
Global map showing the position of poleward mangrove range limits in 21 regions, as identified in four widely used global mangrove distribution maps: the World Atlas of Mangroves (WAM-1) (Spalding et al., 1997), the World Atlas of Mangroves (WAM-2) (Spalding et al., 2010), the Global Distribution of Mangroves (GDM) (Giri et al., 2011) and the Global Mangrove Watch (GMW) (Bunting et al., 2018). Line symbols (grey) denote the position of these poleward range limits as identified by Quisthoudt et al. (2012), which is considered as a reference map in our study.

**Fig. 3:**
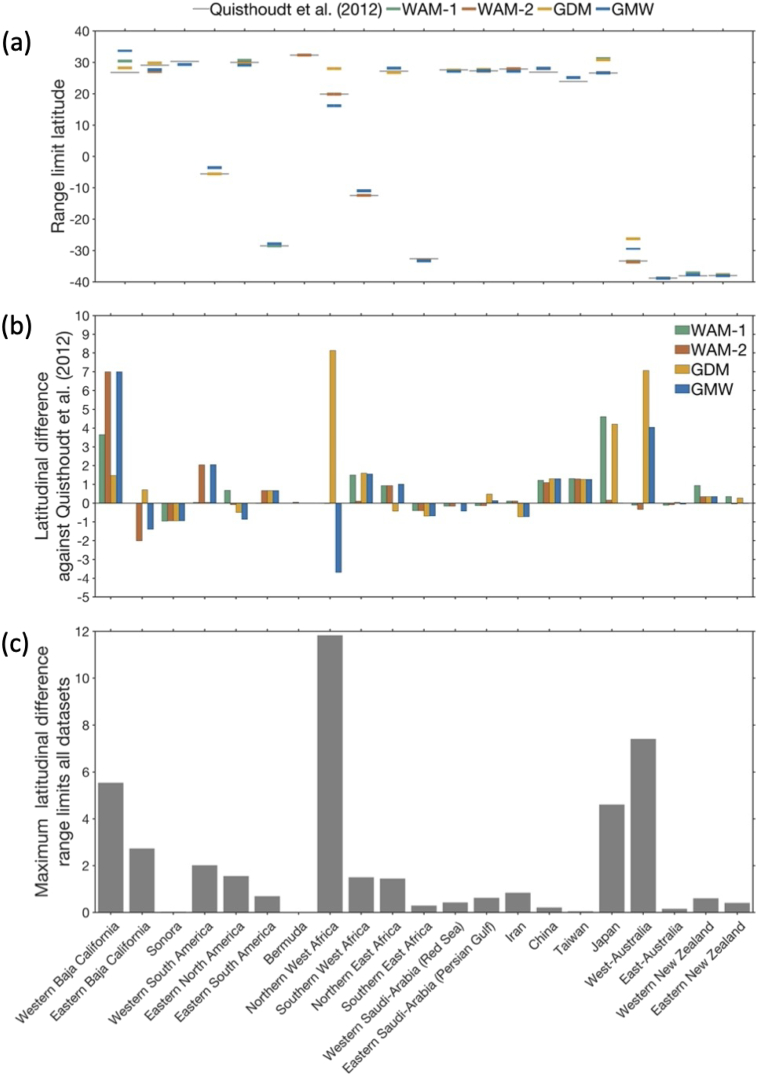
(a) Latitude of poleward mangrove range limits in 21 regions, as identified in four widely used global mangrove distribution maps; (b) Difference in latitudinal position, computed against the latitude of these poleward limits identified by Quisthoudt et al. (2012); (c) Maximum difference in poleward range limit latitude for four widely used global mangrove distribution maps, i.e., not including the Quisthoudt et al. (2012) reference dataset.

Comparison of the global mangrove maps reveals important discrepancies between range limit latitudes (Fig. 2, Fig. 3).

In all global mangrove maps, the southernmost range limit is found in East-Australia (38.84°S ± 0.06°). However, the location of the northernmost range limit differs between the different mangrove maps. While this range limit is found in Bermuda at 32.30°N in the Quisthoudt et al. (2012) data, the northernmost global range limit is found in Japan in the WAM-1 and the GDM maps, at 31.21°N and 30.81°N, respectively, and in California at 33.80°N in the WAM-2 and GMW maps (California mangroves were introduced; see further information in Section 4.1).

The latitudinal difference for range limits between the datasets is <1° for eleven of the 21 range limit areas considered, but exceeds 4°, 5°, 7° and 11° for Japan, Western Baja California, West-Australia, and Northern West Africa, respectively (Fig. 3).

### 3.3 Assessment of bioclimatic data at mangrove range limits

**Fig. 4:**
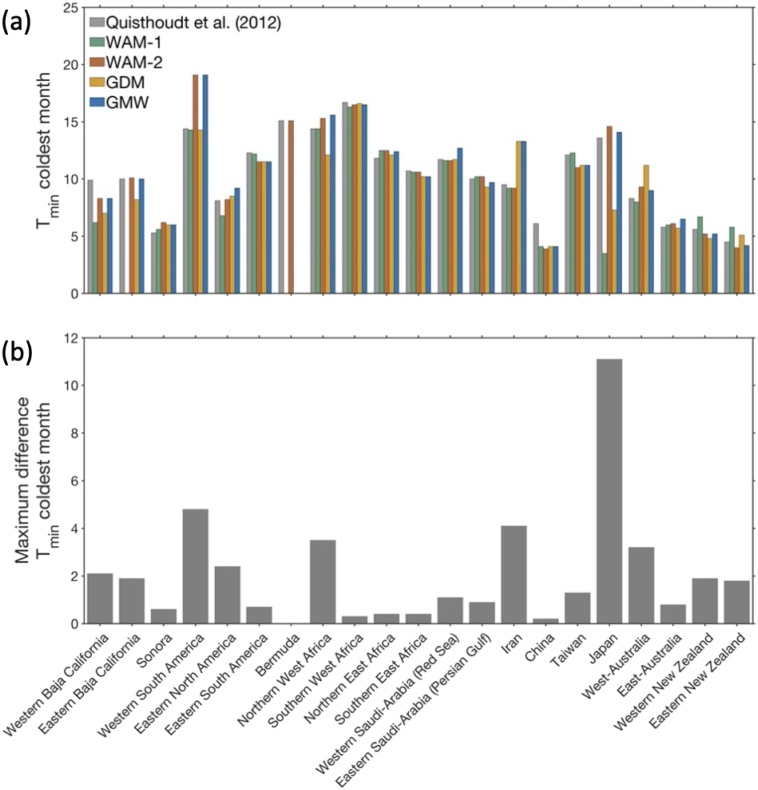
(a) Comparison of minimum air temperature of the coldest month (BIO6) at the poleward range limit positions identified in four widely used mangrove distribution maps and the Quisthoudt et al. (2012) reference data, and (b) maximum difference for this bioclimatic variable between the four widely used global mangrove distribution maps, i.e., not including the Quisthoudt et al. (2012) reference dataset. Environmental data were extracted from the WorldClim v2.1 dataset (Fick and Hijmans, 2017).

**Fig. 5:**
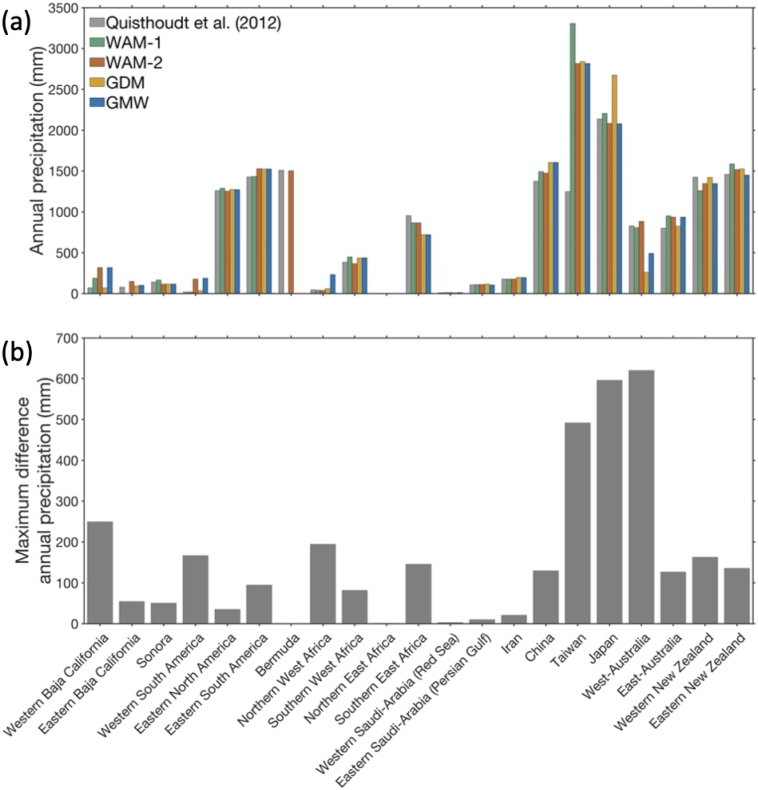
(a) Comparison of annual precipitation (BIO12) at the poleward range limit positions identified in four widely used mangrove distribution maps and the Quisthoudt et al. (2012) reference data, and (b) maximum difference for this bioclimatic variable between the four widely used global mangrove distribution maps, i.e., not including the Quisthoudt et al. (2012) reference dataset. Environmental data were extracted from the WorldClim v2.1 dataset (Fick and Hijmans, 2017).

**Fig. 6:**
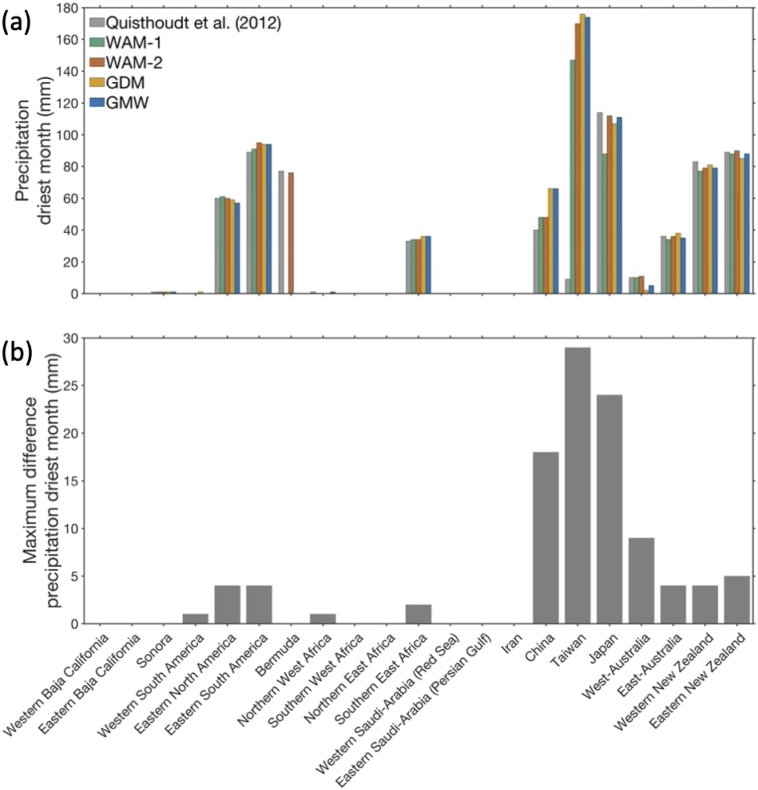
(a) Comparison of precipitation of the driest month (BIO14) at the poleward range limit positions identified in four widely used mangrove distribution maps and the Quisthoudt et al. (2012) reference data, and (b) maximum difference for this bioclimatic variable between the four widely used global mangrove distribution maps, i.e., not including the Quisthoudt et al. (2012) reference dataset. Environmental data were extracted from the WorldClim v2.1 dataset (Fick and Hijmans, 2017).

The largest latitudinal discrepancies found in Japan, Western Baja California, West-Australia, and Northern West Africa, are associated with pronounced differences in minimum temperature of the coldest month (BIO6) of 11.1 °C, 2.1 °C, 3.2 °C, and 3.5 °C (Fig. 4), and differences in annual precipitation of 596 mm, 250 mm, 620 mm, and 195 mm, respectively (Fig. 5). Differences in precipitation of the driest month associated with latitudinal discrepancies are relatively small overall, and is most pronounced for the range limit in China (18 mm), Japan (24 mm) and Taiwan (29 mm) (Fig. 6).

The lowest minimum temperature of the coldest month (BIO6) is found for the WAM-1 dataset (3.5 °C), in Japan, the northernmost global range limit in that dataset, whereas the warmest minimum temperature of the coldest month is found for the Western South America mangrove range limit (19.1 °C) in the WAM-2 and GMW datasets (Fig. 4). For annual precipitation (BIO12), the lowest (3 mm) and highest (3308 mm) values are found for the Northern East Africa range limit in the GDM dataset and the Taiwan range limit in the WAM-1 dataset, respectively (Fig. 5). Precipitation of the driest month (BIO14) was lowest (0 or 1 mm) at the range limits in California, West Africa, Northern East Africa, Saudi Arabia and Iran, in all datasets. The highest value for this environmental variable was found for the range limit in Taiwan in the GDM map (Fig. 6).

## 4 Discussion

Mangrove maps present valuable tools for conservation projects and scientific studies at regional and global scales (Polidoro et al., 2010; Worthington et al., 2020). Although remote sensing techniques are improving rapidly, especially with regards to the implementation of complex algorithms for semi-automatic classification, mapping mangroves at global scale remains a challenging task. Differences in the imagery source and analysis methodologies can lead to discrepancies among maps derived from remote sensing data, algorithm performance and technical evaluation. This has substantial consequences at range limits where detailed maps are most needed. Any failure of globally applicable maps to adequately represent these regions, has substantial consequences for their use in accurately determining their response to climate–based models of changes in extent and structure. The demonstrated variation in range limits between existing global maps is evidence for the potential for error in climatic–based models and the important of accurately representing these small but critical domains.

### 4.1 Mapping introduced mangroves

In some locations, mangroves have been introduced by humans. Some mapping efforts may have the objective to map only the natural occurrences of mangroves, but others may include introduced mangrove areas as well. Except the WAM-1, all maps include the introduced mangroves in Hawaii, USA (Allen, 1998). However, the GDM map is more inclusive than the other maps, also including the introduced mangroves in Morocco (Giri et al., 2011), which leads to a large difference in the location of the northwest Africa mangrove range limit between Giri et al. (2011) and Quisthoudt et al. (2012). Similarly, the WAM-2 and GMW maps include introduced mangroves in San Diego, California (Bardou et al., 2021), which also leads to a more northern range limit for Western Baja California. Distinguishing between natural and introduced mangroves is important to improve the outcome of studies on macroecological processes of dispersal, distribution and expansion.

Since introduced mangroves persist at a site where they are out of their actual range limits, this fact indicates that the climate is appropriate for their survival. Despite suitable climate conditions beyond range limits, the propagule's dispersal is an evident problem, for example, either because of a lack of suitable habitat (due to coastal geomorphology) between the introduced site and the nearest natural mangrove colony or a longshore drift taking propagules away, or a combination of both factors (e.g. Ximenes et al. (2021)).

### 4.2 Mapping mangroves at their range limits

Our comparative analysis of widely used global mangrove maps indicates important discrepancies in the latitudes of the leading edge location of different mangrove range limits globally. We wish to draw attention to the importance of monitoring the expansion and retraction of mangrove range limits since these areas could be considered as sentinel sites to study the impacts of global environmental change on mangrove ecosystems (Quisthoudt et al., 2012; Cavanaugh et al., 2014; Osland et al., 2017b; Ximenes et al., 2018). Yet, whereas satellite images or aerial photographs have been successfully used to map mangroves at local scale focusing on specific areas (Taureau et al., 2019), the urgent need to better understand the worldwide distribution patterns of mangroves makes the production of global mangrove maps crucial. To date, these mangrove range limits have been ignored or mispositioned in some global maps, as observed in eastern South America or western Australia. These errors are largely due to challenges in the identification of the small, sparse mangroves in the satellite imagery used to create global maps.

The Brazilian mangroves limits are ignored by the most recent global mappings with high spatial resolution and powerful classification methods. However, the only mapping that could capture this ecosystem at its limits in Brazil was Spalding et al. (1997), even with older technology than other mappings. For this reason, a very important element is the knowledge of local experts with respect to the range limits, mainly with ground truth data and fieldwork expeditions to the range limits sites.

This is a major limitation since mangrove ecosystems need a consistent policy–supported classification within their geographical range boundaries to enable decision-makers to define policies to preserve and conserve them (Rog and Cook, 2017).

### 4.3 Implications of varying range limits

Mangrove distribution maps can provide valuable insight into the processes and thresholds that control range limits (e.g., Cavanaugh et al. (2015); Osland et al. (2017b). However, uncertainty in the species distribution data used to parameterize species distribution models will result in uncertainty in the output of those models (Luoto et al., 2005). We identified large discrepancies in climatic conditions at some of the range limits across our distribution maps (Fig. 4, Fig. 5). For example, for the poleward mangrove range limit in Japan, there were differences of >600 mm and >10 °C. These differences limit our ability to accurately identify temperature and precipitation thresholds associated with mangrove range limitation. Such knowledge is important to better understand the conditions that allow mangroves to grow, survive, and reproduce, and hence, to forecast potential future range shifts and inform spatial management.

Uncertainty in mangrove distribution data also directly influences the results of predictive climate-driven biomass and soil carbon (C) models. Modeled estimates of global mangrove carbon stocks rely on these distribution data to scale up their estimates of carbon density, e.g., Hutchison et al. (2013); Sanders et al. (2016). As a result, global carbon estimates will only be as good as the underlying estimates of mangrove extent. Uncertainty in these estimates also has subsequent implications for C accounting and the quantification of C offsets. This is critical in an era where nature-based climate solutions are sought, whereby estimates of land cover accounting will require thorough verification. This is visualised in the Dataset Explorer application at www.mangroves4sdgs.com, which compiles all existing global mangrove maps of extent/cover, biomass, and soil carbon, including 3 of the 4 datasets used in this study.

### 4.4 Recommendations

We recognise the difficulties regarding the detection of leading mangroves edges as the structure and areal mangrove extent at these sites is generally small and hence, difficult to capture with most methodologies used to map mangrove forests at large spatial scales (e.g., global). In short, we give some recommendations to overcome this issue.

For the next mangrove mapping generation, we propose five recommendations inspired by Congalton et al. (2014) and Grekousis et al. (2015) who reviewed a large number of regional and global land cover maps.

Firstly, future mangrove mapping efforts should provide explicit definitions of mangrove classes to the end-users. Any remote sensing-based classification is impacted by the semantic gap issue, i.e., the lack of agreement between the information that one can extract from the visual data and the interpretation made of the same data by a user in a given situation (Smeulders et al., 2000). In other words, there is a gap between the low-level information contained in multi-spectral signatures and clustered by automatic classification algorithms and the high-level semantic interpretation made by an end-user. The point here is not necessarily to map additional mangrove types to achieve finer maps but to clarify what the map producer has considered as ‘mangrove’. For example, it is sometimes unclear if introduced mangroves are considered in global maps or if urban mangroves have been discarded.

Secondly, it is important to further improve the spatial and temporal resolution of the global mangrove maps. According to Grekousis et al. (2015), global land cover maps should be released at 10 m to 30 m spatial resolution at least every five years. In this regard, the implementation of new global maps based on Sentinel-1 and Sentinel-2 data, combining radar and optical imagery at 10 m and with a high temporal resolution appears promising. Moreover, new global products could be beneficial to assess mangrove seasonal patterns based on a Sentinel-2 time series, as illustrated for example for the state of Sinaloa, Mexico (Valderrama-Landeros et al., 2021). This point appears particularly important to map the expansion or retraction of mangrove range limits of which the importance has been emphasized earlier in this paper. However, 10 m imagery may still not be sufficient to map some of the small mangroves found at range limits, and so higher resolution aerial and satellite imagery should be used for select locations.

Thirdly, the methodological approaches need to be well documented and transparent in order to facilitate comparisons with other maps (Congalton et al., 2014). In the case of the global mangrove maps compared in this study, manual edition based on visual interpretation (as in WAM-1) turns the method operator-dependent and subjective, thus difficult to describe to the end-users. On the other hand, maps based on data-driven approaches using advanced supervised and unsupervised classification algorithms (as in GDM and GMW) are easier to describe. Moreover, machine learning algorithms should be taken into consideration for mangrove mapping (Pham et al., 2019). However, these approaches depend on the training and validation datasets, which is another point discussed by Grekousis et al. (2015) who recommends improving the collection of training and validation datasets. In addition, limitations of the imagery and methodology should be clearly described. For example, it may not be possible to detect the small-stature mangroves found near many poleward range limits using moderate resolution imagery (10–30 m).

Fourthly, refers to map accuracy. Global maps usually suffer from spatial heterogeneity, especially when training samples used in supervised classifiers are geographically unequally distributed. To overcome this issue, Grekousis et al. (2015) recommends developing pixel-based accuracy metrics. For instance, when using supervised classifiers, this could be done by releasing probabilities of the mangrove class to which the pixels belong.

The research community must be aware of the limitations of their datasets before using them in additional studies. It is important that sources of error and uncertainty are understood and propagated appropriately in order to avoid the inappropriate use of a dataset or provide recommendations based on results which may have built–in yet hidden error. An understanding of such limitations and therefore appropriate use of the global maps will improve transparency in derived products.

Finally, it is worth noting that the emergence of online platforms to process big Earth Observation data, such as Google Earth Engine (GEE), may help address most of these recommendations by facilitating the sharing of transparent and reproducible processing chains (Gorelick et al., 2017). For example, Diniz et al. (2019) used the GEE to compute the annual status of Brazilian mangroves from 1985 to 2018 based on the automatic computation of a new Modular Mangrove Recognition Index (MMRI) applied on Landsat images.

## 5 Conclusions

Accurately mapping mangroves at their range limits is important since these locations are likely to be especially sensitive to climate change. We conclude that the four global mangrove maps have little consensus on the location of mangrove range limits. Here, we show that in at least 10 mangrove range limit regions globally the position of the poleward range boundary differs for >1° in latitude between the four widely used global mangrove distribution products considered in this study. Dissimilarities in mapping mangrove range limits can jeopardise investigations of the sensitivity of range limits to climate variability, predictions of range dynamics and future range shifts, assessments of biomass and carbon stocks, and estimates of deforestation rates. Future mapping efforts should give more attention to accurately characterise the position of range edges, ideally combining data from field-based surveys, local expert knowledge, and very high-resolution observations, such as sub-metric satellite imagery and/or LiDAR sensors mounted on drones, while being considerate of the detection errors associated with each survey methodology. A more accurate representation of range limits will contribute to better predicting mangrove range dynamics and shifts in response to climate change.

## Funding

ACX was supported by the Brazilian scholarship Science without Borders from the 10.13039/501100003593National Council for Scientific and Technological Development (CNPq) (201782/2014-6). T.V.d.S. was supported by the EU 10.13039/100010661Horizon 2020 Framework Programme for Research and Innovation under the Marie Skłodowska-Curie actions Individual Fellowship (MSCA-IF) with grant agreement No 896888 (GLOMAC). DM was supported by the 10.13039/100000200United States Agency for International Development (USAID) Grant No MTO 069033.

## CRediT authorship contribution statement

**Arimatéa C. Ximenes**: Conceptualization, Methodology, Validation, Formal Analysis, Investigation, Data Curation, Writing - Original Draft, Reviewing and Editing, Visualization, Funding acquisition. **Kyle C. Cavanaugh:** Writing - Reviewing and Editing, Methodology, Visualization, Validation. **Damien Arvor:** Writing - Reviewing and Editing. **Daniel Murdiyarso:** Writing - Reviewing and Editing, Funding acquisition. **Nathan Thomas:** Writing - Reviewing and Editing. **Gustavo** **F.B.** **Arcoverde:** Writing - Reviewing and Editing. **Polyanna da Conceição** **Bispo:** Writing - Reviewing and Editing. **Tom Van der Stocken:** Conceptualization, Methodology, Validation, Formal Analysis, Investigation, Data Curation, Writing - Reviewing and Editing, Visualization.

## Declaration of competing interest

The authors declare that they have no known competing financial interests or personal relationships that could have appeared to influence the work reported in this paper.

## Data Availability

All data used in this research is freely available online and it is said in the paper.
